# Hypersensitivity reaction studies of a polyethoxylated castor oil-free, liposome-based alternative paclitaxel formulation

**DOI:** 10.3892/mmr.2013.1264

**Published:** 2013-01-04

**Authors:** HONGBO WANG, GUANG CHENG, YUAN DU, LIANG YE, WENZHONG CHEN, LEIMING ZHANG, TIAN WANG, JINGWEI TIAN, FENGHUA FU

**Affiliations:** 1 Key Laboratory of Molecular Pharmacology and Drug Evaluation (Ministry of Education of China), School of Pharmacy, Yantai University, Yantai 264005; 2 R&D Center, Nanjing Luye Sike Pharmaceutical Co., Ltd, Nanjing 210061; 3 State Key Laboratory of Long-Acting and Targeting Drug Delivery Technologies (Luye Pharma Group Ltd.), Yantai 264003, P.R. China

**Keywords:** liposome, Lipusu, Taxol, complement activation, hypersensitivity reaction

## Abstract

The commercial drug paclitaxel (Taxol) may introduce hypersensitivity reactions associated with the polyethoxylated castor oil-ethanol solvent. To overcome these problems, we developed a polyethoxylated castor oil-free, liposome-based alternative paclitaxel formulation, known as Lipusu. In this study, we performed *in vitro* and *in vivo* experiments to compare the safety profiles of Lipusu and Taxol, with special regard to hypersensitivity reactions. First, Swiss mice were used to determine the lethal dosages, and then to evaluate hypersensitivity reactions, followed by histopathological examination and enzyme-linked immunosorbent assays (ELISAs) of serum SC5b-9 and lung histamine. Additionally, healthy human serum was used to analyze *in vitro* complement activation. Finally, an MTT assay was used to determine the *in vitro* anti-proliferation activity. Our data clearly showed that Lipusu displayed a much higher safety margin and did not induce hypersensitivity or hypersensitivity-related lung lesions, which may be associated with the fact that Lipusu did not activate complement or increase histamine release *in vivo*. Moreover, Lipusu did not promote complement activation in healthy human serum *in vitro*, and demonstrated anti-proliferative activity against human cancer cells, similar to that of Taxol. Therefore, the improved formulation of paclitaxel, which exhibited a much better safety profile and comparable cytotoxic activity to Taxol, may bring a number of benefits to cancer patients.

## Introduction

Paclitaxel, which was isolated from *Taxus brevifolia* in the early 1970s and approved by the FDA in 1993, is one of the most active antineoplastic agents against a wide spectrum of malignancies, including ovarian, breast, lung, head and neck cancers, and Kaposi’s sarcoma (1,2). Due to its poor solubility, conventional preparations of paclitaxel (Taxol) were formulated in a special vehicle, which contained polyethoxylated castor oil (PCO) and anhydrous ethanol (1:1 V/V) (3–5). Despite the extensive clinical utilization and success of Taxol, serious toxic effects, such as serious hypersensitivity reactions and neurotoxicity, are associated with PCO (4–6). To prevent and manage these serious problems, premedication with high doses of corticosteroids and antihistamines must routinely be administrated (6), which increases the possibility of drug interactions. Therefore, an improved paclitaxel formulation, which could eliminate the adverse effects associated with PCO while retaining similar anticancer activity, would greatly benefit cancer patients and their caregivers.

Surfactant-free paclitaxel formulations, which could replace Taxol, have been the object of considerable investigation (7). To date, promising alternative formulations involved the use of albumin nanoparticles, polyglutamates, taxane analogs, prodrugs, emulsions and liposomes (8,9). Among those, only one paclitaxel protein-bound particle for injection (Abraxane) has been approved by the FDA for the treatment of breast cancer (10), and several novel formulations with different vehicles are at different stages of preclinical studies or clinical trials (11,12).

Among all drug carrier systems, liposomes represent a mature, versatile technology with considerable potential for encapsulation of lipophilic and hydrophilic drugs (8), and have been used for the treatment of neoplastic and infectious diseases, such as injectable doxorubicin hydrochloride liposome (Doxil) (13) and amphotericin B liposome (Ambisome) (14). Currently, several liposomal PTX formulas are in various stages of clinical trials. A liposome-entrapped paclitaxel formulation (LEP-ETU) (15) and an ionically charged paclitaxel-lipid complex (EndoTAG-1) (16) are in phase II clinical trials. As part of the continuing effort to improve the utilization of paclitaxel-based chemotherapy, we developed a polyethoxylated castor oil-free, liposome-based alternative paclitaxel formulation known as Lipusu, which was launched in China (8). In the current study, we performed *in vitro* and *in vivo* strategies to compare the safety profiles of Taxol and Lipusu, with special regard to the induction of the hypersensitivity reaction.

## Materials and methods

### Materials

Taxol, in which paclitaxel was formulated in PCO and anhydrous ethanol, was purchased from Haikou Pharm (Cat. no.: 111006; Haikou, China). Lipusu, in which paclitaxel was formulated into liposomes, was provided by Luye Pharm (Cat. no.: 20120102; Nanjing, China). In *in vitro* and *in vivo* experiments, Taxol and Lipusu were diluted into the required concentration using 5% glucose according to the manufacturer’s instructions.

### Cell lines and cell culture

The human oral carcinoma cell line KB was purchased from Cell Culture Center of Institute of Basic Medical Sciences, Chinese Academy of Medical Sciences (Beijing, China). The cells were cultured in DMEM media supplemented with 10% fetal calf serum, penicillin (100 U/ml) and streptomycin (10 μg/ml; Gibco BRL, NY, USA), and incubated at 37°C in a humidified air atmosphere containing 5% CO_2_. All cells were harvested in their exponential growth phase.

### Animals

Swiss mice (18–22 g) were obtained from Shandong Luye Pharmaceutical Company (Yantai, China). The animals were housed in a light- and temperature-controlled room (20–24°C, humidity 40–65%) and kept on a standard diet and provided with water. All experiments were performed according to the Guidelines for Care and Use of Experimental Animals of the Experimental Animal Research Committee of Yantai University (China).

### Acute toxicity study

The mice were randomly divided into 10 groups (5/sex/group). Different concentrations of Taxol and Lipusu solution were obtained using geometric dilution with 5% glucose injection at a dose-ratio of 1:0.85. Five doses, 96.8, 82.3, 69.9, 59.4 or 50.5 mg/kg, for Lipusu, and five doses, 44.1, 37.5, 31.8, 27.0 or 23.0 mg/kg, for Taxol, were administered intravenously (i.v.) in a 10 ml/kg injection volume. General behavior was observed continuously for 1 h following treatment; animals were further observed for up to 14 days following treatment for signs of toxicity, mortality and latent time to mortality. The median lethal dose (LD_50_) and 95% confidence limit were determined using the Bliss method (17).

### Anaphylaxis study

Taxol and Lipusu were freshly prepared according to the manufacturer’s instructions and diluted with 5% glucose injection prior to use. The vehicle used as control (5% glucose injection) or the drugs being evaluated (30 mg/kg) were administered i.v. in a 10 ml/kg injection volume to the male mice (8/group) with or without dexamethasone (8 mg/kg, gavage) and cimetidine (40 mg/kg, i.v.) treatment (18). Allergic signs were observed and scored according to Table I. Blood was sampled under anesthesia by inhaling ether 90 min after the single dose, and the animals were then sacrificed by cervical dislocation. Serum was prepared by centrifugation and the content of serum complement split product SC5b-9 was detected using commercial ELISA kits (Jingke Bio, Shanghai, China), according to the manufacturer’s instructions. After the mice were sacrificed, lungs were removed quickly and weighed for the calculation of organ/body ratio. Half of the tissues were cooled, homogenized, and tested for histamine using commercial ELISA kits (Jingke Bio). The remaining lung tissue was used for histological examination, in which all specimens were analyzed and photographed by two pathologists in a blind investigation.

### In vitro complement activation study

Human blood was drawn from 10 healthy volunteers according to protocols approved by the Human Use Committee of Yantai University, and all subjects gave written informed consent to use their blood for research purposes. Specimens were incubated with the drugs being evaluated at the volume ratio of 3:1, as previously reported with minor modifications (5). Briefly, 5 μl of drugs being evaluated with a concentration of 1 mg/ml were mixed with 15 μl serum in Eppendorf tubes and incubated in a shaking table (80 rpm cycle) at 37°C for 60 min. The reaction was stopped by adding 980 μl PBS with 10 mM EDTA (pH 7.4), and the SC5b-9 content was determined with commercial ELISA kits (Jingke Bio).

### In vitro cytotoxicity assay

The cytotoxicity of Taxol and Lipusu were determined by MTT assay, as described previously with minor modifications (19). Briefly, KB cells were seeded into a 96-well plate at 4,000 cells per well. Culture medium was then replaced with 200 μl medium containing serial dilutions of the drugs. After 72 h of incubation at 37°C, MTT stock solution (500 μg/ml) was added into each well, and the plate was incubated for 2 h. Medium was then removed and DMSO was added to dissolve formazan crystals converted from MTT. Cell viability was assessed by absorbance at 570 nm measured using a microplate reader (Wellscan MK3, Helsinki, Finland).

### Data analysis and statistics

Results are presented as the means ± SD. Comparisons between more than two groups used analysis of variance (one way ANOVA), followed by the Student’s t-test. P≤0.05 was considered to indicate a statistically significant difference.

## Results

### Lipusu exhibited a greater safety margin than Taxol

Single dose acute toxicity assays were performed on Swiss mice for Taxol or Lipusu, and the mortalities and clinical signs were observed. The majority of the Taxol-injected animals were subsequently demonstrated anaphylactic responses such as piloerection, anhelation and syncope, which were not observed in the Lipusu-injected animals. The mortality of these animals was recorded from the dosing day (Day 1) until the end of observation (Day 14), and the mortalities for Taxol and Lipusu are shown in Table II. Based on these results, the LD_50_ values (95% confidence limits) for Lipusu and Taxol were calculated to be 69.82 mg/kg (58.9–82.7) and 33.0 mg/kg (30.2–36.1), respectively.

### Lipusu induced much milder hypersensitivity reactions in mice than Taxol

Taxol or Lipusu, at a dosage of 30 mg/kg, were intravenously injected into mice. Behaviors were observed and hypersensitivity reactions were ranked according to Table I. The animals in the Taxol group were all observed to have acute hypersensitivity reactions at 2–5 min after injection, and recovered 17–30 min later. All the animals in the Lipusu group, however, showed much milder reactions. The severity of the hypersensitivity reactions induced by paclitaxel injection, particularly for Taxol, could be attenuated by pretreatment with dexamethasone and cimetidine. The individual response for each animal was summarized in Table III.

### Lipusu did not induce pulmonary edema

**Figure 1:**
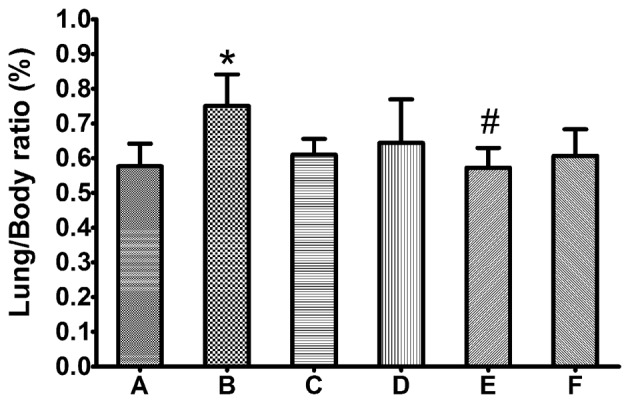
Effects of Lipusu and Taxol in the absence or presence of premedication on the lung/body ratio. The lungs were harvested and weighted 90 min after i.v. administration with 5% glucose (A or D), Taxol (B or E) and Lipusu (C or F) in the absence or presence of dexamethasone and cimetidine. All data are expressed as the means ± SD (n=8). ^*^P<0.05, compared with the 5% glucose group; ^#^P<0.05, compared with Taxol in the absence of dexamethasone and cimetidine group.

**Figure 2:**
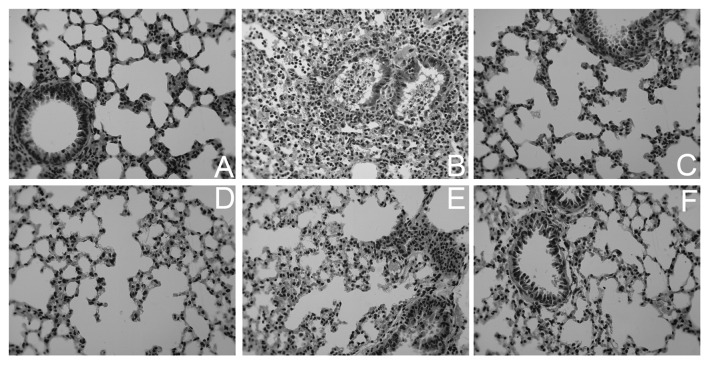
Effects of Lipusu and Taxol in the presence or absence of premedication on the histopathological changes in the lung. Animal tissue was fixed and hematoxylin and eosin (HE) staining was performed. Light microscope observations were carried out, and the representive images are shown. Groups are the same as Fig. 1. (magnification, ×400).

Lungs were harvested and weighed 90 min after injection of the drugs or the control vehicle solution, after which the lung weight/body weight ratio (%) was calculated. As shown in Fig. 1, the lung/body ratio was significantly increased in animals treated with Taxol compared with the control group (P≤0.05), whereas there was no significant difference between the control and Lipusu groups. By histological examination, more severe lung injuries were observed in Taxol-treated animals, including pulmonary edema, infiltration of tissue, alveoli with inflammatory cells and signs of tissue injury, and thickening of alveolar walls (Fig. 2). The histological appearance of lungs in Lipusu-treated animals, however, was relatively normal. Pretreatment with dexamethasone and cimetidine greatly attenuated the lung damage in Taxol-treated mice.

### Lipusu did not induce complement activation or increase histamine accumulation

**Figure 3:**
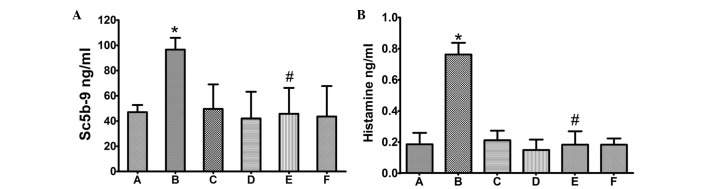
Effects of Lipusu and Taxol in the presence or absence of premedication on the serum complement activation and lung histamine release. The bloods were sampled and the lung tissues were homogenized, and then the content of serum SC5b-9 and lung histamine were detected by commercial ELISA kits. Groups are the same as Fig. 1. All data are expressed as the means ± SD (n=8). ^*^P<0.05, compared with 5% glucose group; ^#^P<0.05, compared with Taxol in the absence of dexamethasone and cimetidine group.

Serum SC5b-9 content was measured by ELISA. As shown in Fig. 3, SC5b-9 was significantly induced in animals administered Taxol (P≤0.05, compared with control group), but not in animals administered the same dosage of Lipusu. Notably, the increased serum SC5b-9 was markedly ameliorated by pretreatment with dexamethasone and cimetidine (P≤0.05, compared with the Taxol group), which are glucocorticoid and histamine H2-receptor antagonists, respectively. Similar results were observed for lung histamine (detected by ELISA); administration of Taxol, but not Lipusu, increased the content of histamine in lung tissue (Fig. 3), which could also be blocked significantly by premedication.

### Lipusu did not induce complement activation in vitro in healthy human serum

**Figure 4:**
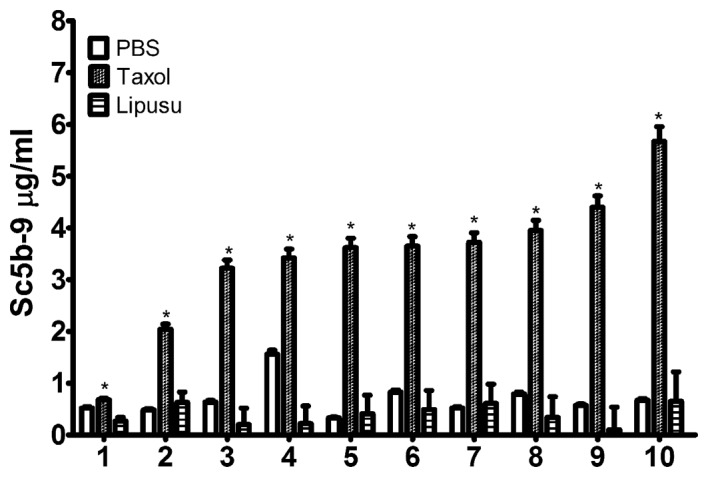
Effects of Lipusu and Taxol on SC5b-9 formation in healthy volunteer sera. Taxol, Lipusu or a corresponding volume of PBS was incubated with serum samples from 10 healthy volunteers for 30 min, and the content of SC5b-9 was determined by a commercial ELISA kit. All data are expressed as the means ± SD (n=3). ^*^P<0.05, compared with PBS group.

The effects of Taxol and Lipusu on complement activation were determined *in vitro* using sera from healthy subjects. Following incubation with the drugs, serum SC5b-9 content was quantified using an ELISA kit. As shown in Fig. 4, Taxol significantly increased the amount of terminal complement activation products in all 10 normal subjects following incubation for 30 min at 37°C, which was not observed in sera after incubation with either 5% glucose injection or with Lipusu. Our results also showed differences in complement response to Taxol in different subjects, as changes in SC5b-9 levels ranged from 2-fold to 6-fold in various individuals.

### Lipusu and Taxol showed similar cytotoxic activity against KB cancer cells in vitro

**Figure 5:**
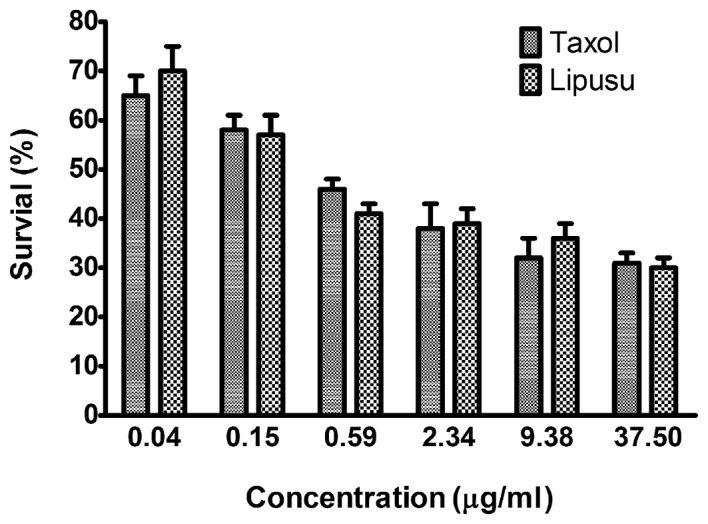
Effects of Lipusu and Taxol on cell viability *in vitro*. KB cells were seeded into a 96-well plate and treated with Taxol or Lipusu at the desired concentration. The cell viability was detected by MTT assay after 72 h incubation. All data are expressed as the means ± SD (n=3).

*In vitro* cytotoxicity of Lipusu against KB oral carcinoma cells was compared with that of Taxol using an MTT assay. To achieve different doses of paclitaxel, standard formulations of Taxol and Lipusu were diluted with the culture media, resulting in the final concentrations (0.04–37.5 μg/ml). As shown in Fig. 5, Taxol and Lipusu displayed robust anti-proliferation activity against KB cancer cells in a dose-dependent manner, with no significant difference between these two formulations.

## Discussion

Paclitaxel is a powerful anti-cancer drug and is used widely against several types of malignant tumors (12). Due to its poor solubility, commercial paclitaxel is prepared with a PCO-ethanol solvent, which introduces serious side effects, such as hypersensitivity and neurotoxicity (8). To overcome these problems, several strategies have been used to improve paclitaxel formulations without PCO (7). In this study, we reported for the first time a novel paclitaxel-liposome formulation designated as Lipusu, which was proven to eliminate the hypersensitivity reactions while retaining robust anti-proliferative activity similar to Taxol.

The LD_50_ for the two formulations was first determined in Swiss mice. Lipusu, with a higher LD_50_, was shown to have a greater safety margin than Taxol. As the PCO and ethanol vehicle in Taxol is considered to be the toxic agent and main cause of hypersensitivity (15), Lipusu, which is devoid of PCO and ethanol, was observed to have greatly decreased overall toxicity. Above all, the anaphylaxis-like symptoms associated with PCO, such as syncope and dyspnea, were only observed in animals injected with Taxol, but not in those injected with Lipusu.

An anaphylaxis study was then performed using bioequivalent dosages to those used in the clinic. Almost all of the Taxol-injected mice were observed to have different degrees of hypersensitivity reactions, which were not observed in Lipusu-injected animals. Based on the asthma-like findings in the animals with hypersensitivity reactions, the lungs were weighed and examined. Significantly increased lung/body ratios and accumulation of inflammatory exudate were common in animals injected with Taxol instead of Lipusu, which may be responsible for the respiratory symptoms in animals. Based on our unpublished data, both Taxol and Lipusu were quickly redistributed to the lung post-injection, in which the inflammatory cells and exudate accumulated, resulting in the symptoms described above.

Unwanted complement activation played an important role in Taxol-induced hypersensitivity and tissue lesions (5,6). After systemic exposure to Taxol rather than Lipusu, the animals were observed to have increased serum SC5b-9, which may be initiated by PCO binding with C3 (20). As the terminal product of complement activation, it could directly bind with membranes and cause osmatic lysis of target cells (20,22). The active molecules produced during complement activation, such as C3a and C5a, could bind to mast cells to trigger histamine release (6), which consequently increased microvascular permeability and promoted the exudation of inflammation cells (21). The pathophysiologic changes resulting from increased SC5b-9 and aggregated histamine contributed to the clinical signs as well as the hypersensitivity reactions. Notably, the hypersensitivity reactions and the lung lesions, as well as the increased content of serum SC5b and lung histamine, were alleviated significantly by pretreatment with corticosteroids and antihistamines, as used in clinics.

To further confirm the anaphylaxis findings in animal experiments, sera from healthy volunteers were used to perform the *in vitro* complement activation assay. Consistent with the literature (5,6), Taxol was observed to activate complement *in vitro*, which was indicated by the increased SC5b-9 content. Lipusu incubated at same concentration, however, could not activate the complement pathway. These findings, with regard to the activity of Lipusu on complement activation, were not completely consistent with certain reports (20), in which it was shown that liposome-based formulations induced hypersensitivity reactions. Other researchers, however, published similar findings to ours, in which liposome-based paclitaxel did not induce hypersensitivity reactions (23,24). The exact circumstances and mechanisms in which hypersensitivity reactions were induced by liposome formulations need to be further explored.

The last question addressed in this study called for comparison of the antitumor activities of these two formulations. From the *in vitro* data, no significant difference in cytotoxic activity was observed between these two formulations by MTT assay in KB cells. Together with the results mentioned above, Lipusu did not induce hypersensitivity reactions *in vitro* and *in vivo*, and retained robust anti-proliferative activity, which would improve the compliance of cancer patients.

In conclusion, Lipusu, a novel liposome-based paclitaxel formulation, was proven to eliminate the hypersensitivity reactions associated with PCO, while showing anti-proliferative activity similar to Taxol. Therefore, the improved formulation of paclitaxel will bring a number of benefits to cancer patients.

## Figures and Tables

**Table I:** The severity of hypersensitivity reactions.

Grade	Clinical signs
0/−	Normal
1/+	Disturbance, head shaking
2/++	Shortness of breath, drowsiness
3/+++	Dyspnea, syncope, gatism
4/++++	Mortality

**Table II:** The mortality and clinical signs of animals following injection with Taxol and Lipusu in the acute toxicity study.

Drug	Formulation (paclitaxel, mg/kg)	Animals/group	Mortalities/group	Mortality ratio (%)	Clinical signs
Lipusu	96.8	10	8	80	Asthenia, anorexia
	82.3	10	6	60	Asthenia, anorexia
	69.9	10	5	50	Asthenia, anorexia
	59.4	10	3	30	None
	50.5	10	3	30	None
Taxol	44.1	10	9	90	Asthenia, anorexia, syncope, dyspnea
	37.5	10	7	70	Asthenia, anorexia, syncope, dyspnea
	31.8	10	5	50	Asthenia, anorexia, syncope, dyspnea
	27.0	10	2	20	Asthenia, anorexia, syncope, dyspnea
	23.0	10	0	0	Asthenia, anorexia, syncope, dyspnea

**Table III:** The rank of hypersensitivity reactions for the animals after injection with Taxol or Lipusu at the therapeutic dosage.

No.	5% glucose	Taxol	Lipusu	5% glucose premedication	Taxol premedication	Lipusu premedication
1	−	+++	+	−	+	+
2	−	+++	+	−	+	−
3	−	+++	+	−	++	+
4	−	+++	+	−	+	+
5	−	+++	++	−	+	+
6	−	+++	+	−	+	−
7	−	+++	+	−	++	+
8	−	+++	+	−	++	+
